# Optimization of Multi-Generation Multi-location Genomic Prediction Models for Recurrent Genomic Selection in an Upland Rice Population

**DOI:** 10.1186/s12284-023-00661-0

**Published:** 2023-09-27

**Authors:** Hugues de Verdal, Cédric Baertschi, Julien Frouin, Constanza Quintero, Yolima Ospina, Maria Fernanda Alvarez, Tuong-Vi Cao, Jérôme Bartholomé, Cécile Grenier

**Affiliations:** 1 CIRAD, UMR AGAP Institut, 34398 Montpellier, France; 2 UMR AGAP Institut, Univ Montpellier, CIRAD, INRAE, Institut Agro, 34398 Montpellier, France; 3 Alliance Bioversity-CIAT, A.A.6713, Km 17 Recta Palmira Cali, Cali, Colombia

**Keywords:** Training set optimization, Genomic selection, *Oryza sativa*, Genotype-by-environment interaction

## Abstract

Genomic selection is a worthy breeding method to improve genetic gain in recurrent selection breeding schemes. The integration of multi-generation and multi-location information could significantly improve genomic prediction models in the context of shuttle breeding. The Cirad-CIAT upland rice breeding program applies recurrent genomic selection and seeks to optimize the scheme to increase genetic gain while reducing phenotyping efforts. We used a synthetic population (PCT27) of which S_0_ plants were all genotyped and advanced by selfing and bulk seed harvest to the S_0:2_, S_0:3_, and S_0:4_ generations. The PCT27 was then divided into two sets. The S_0:2_ and S_0:3_ progenies for PCT27A and the S_0:4_ progenies for PCT27B were phenotyped in two locations: Santa Rosa the target selection location, within the upland rice growing area, and Palmira, the surrogate location, far from the upland rice growing area but easier for experimentation. While the calibration used either one of the two sets phenotyped in one or two locations, the validation population was only the PCT27B phenotyped in Santa Rosa. Five scenarios of genomic prediction and 24 models were performed and compared. Training the prediction model with the PCT27B phenotyped in Santa Rosa resulted in predictive abilities ranging from 0.19 for grain zinc concentration to 0.30 for grain yield. Expanding the training set with the inclusion of the PCT27A resulted in greater predictive abilities for all traits but grain yield, with increases from 5% for plant height to 61% for grain zinc concentration. Models with the PCT27B phenotyped in two locations resulted in higher prediction accuracy when the models assumed no genotype-by-environment (G × E) interaction for flowering (0.38) and grain zinc concentration (0.27). For plant height, the model assuming a single G × E variance provided higher accuracy (0.28). The gain in predictive ability for grain yield was the greatest (0.25) when environment-specific variance deviation effect for G × E was considered. While the best scenario was specific to each trait, the results indicated that the gain in predictive ability provided by the multi-location and multi-generation calibration was low. Yet, this approach could lead to increased selection intensity, acceleration of the breeding cycle, and a sizable economic advantage for the program.

**Supplementary Information:**

The online version contains supplementary material available at 10.1186/s12284-023-00661-0.

## Introduction

Several studies have demonstrated empirically or by simulation the value of genomic selection (GS) for crop breeding in wheat (Crossa et al. 2010; Heffner et al. 2011; Rutkoski et al. 2012), maize (Bernardo and Yu 2007; Zhao et al. 2012; Crossa et al. 2013), barley (Lorenz et al. 2012a; Endelman et al. 2014; Sorrells 2015) or rice (Onogi et al. 2015; Isidro et al. 2015; Spindel et al. 2015; Grenier et al. 2015; Wang et al. 2017; Ben Hassen et al. 2018b; Bhandari et al. 2019; Ahmadi et al. 2020). Regardless of the trait and species considered, the predictive ability (PA), i.e., the estimated correlation between the phenotypic performances and the predicted values, would allow GS to return higher genetic gain than the classical selection based on phenotypes and pedigree relationship. The ways in which GS can increase genetic gain over a breeding program based on phenotypic selection are numerous (Rutkoski et al. 2017; Crossa et al. 2017; Cobb et al. 2019; Bartholomé et al. 2022). Almost all the parameters of the breeder's equation can be improved with GS. Predicting the value of genotypes using the genomic information acquired on a large number of non-phenotyped entries can significantly impact the breeding program by cutting down the phenotyping effort, but also by increasing selection intensity (R2D2 Consortium et al. 2021). GS can also shorten the breeding cycle length by reducing generation interval (Heffner et al. 2010; Spindel and Iwata 2018; Dreisigacker et al. 2021). However, only a few empirical studies report germplasm development based on GS of promising lines in the early steps of breeding, when heterozygosity levels are high (Mendonça et al. 2020).

Greater precision in the predictions directly affects genetic gain (Falconer and MacKay 1996). Therefore, even a small improvement in PA can have a consequent impact in terms of genetic gain (Xu et al. 2020, 2021). It has previously been shown that PA can be improved when multi-environment data are pooled, and an appropriate model capturing genotype-by-environment interactions (G × E) was used for prediction (Burgueño et al. 2012; Lopez-Cruz et al. 2015; Crossa et al. 2016; Cuevas et al. 2016, 2017; Ben Hassen et al. 2018a; Jarquín et al. 2020; Xu et al. 2021). Multi-environment trials (MET) are commonly performed in plant breeding to evaluate genotypes under different growing conditions and capture G × E. In this context, the use of sparse testing methods in which all the genotyped individuals are phenotyped in at least one environment is attractive in reducing the phenotyping efforts for MET (Jarquín et al. 2017, 2020). Although very promising, this strategy, which relies on the use of various testing locations to calibrate the predictive model, thus accounting for the G × E, is mainly dependent on the level of correlation between locations. MET can be composed of data from various years and locations, but potentially also include testing material from different generations of germplasm during its development. While the ultimate goal of sparse testing is to reduce phenotyping effort in the context of G × E, PA can also vary according to the effects of location, year and generation of phenotyped material. Using genomic information from the most recent or a more homozygous generation in the training set (TS) can impact the PA of GS (Sallam et al. 2015). These authors highlighted the fact that more generations of selfing result in an increased percentage of fixed markers, thus losing their PA.

Another aspect to consider is the relationship between training and validation populations. Optimizing the training population has previously been shown to improve the PA of GS models (Rincent et al. 2012; Isidro et al. 2015; Akdemir et al. 2021a; Rio et al. 2022). Several methods have been developed to optimize selection of the TS based on the relationship between genotypes in the TS and/or between training and validation sets. The selection of genotypes to be phenotyped and included in the TS has two major interests: it could reduce the number of entries to be phenotyped and increase PA.

In addition to improving the breeder's equation components, a gain over conventional marker-assisted selection or over the phenotypic selection is achieved by the use of GS (Heffner et al. 2010; Lorenz et al. 2012b; Ben-Sadoun et al. 2021).

In the case of rice, the potential of GS to accelerate genetic gain has previously been highlighted (Onogi et al. 2015; Isidro et al. 2015; Spindel et al. 2015; Grenier et al. 2015; Wang et al. 2017; Ben Hassen et al. 2018b; Spindel and Iwata 2018; Bhandari et al. 2019; Ahmadi et al. 2020; Bartholomé et al. 2022). The main observations extracted from a review of GS applied to rice (Ahmadi et al. 2020) were that marker set size does not have to be large (Spindel et al. 2015; Bhandari et al. 2019), the population structure needs to be accounted for (Isidro et al. 2015; Grenier et al. 2015; Ben Hassen et al. 2018b), and the relatedness between the TS and the breeding population remains essential to ensure high PA. GS models in rice breeding have been applied to various breeding materials and notably to synthetic populations (Grenier et al. 2015; Morais Júnior et al. 2018a; Baertschi et al. 2021). In those later studies, the PA of the genomic prediction (GP) models evaluated by cross-validation (CV) revealed the potential of the methods to accelerate the genetic gain in recurrent selection (RS) with a particular interest in accounting for the G × E effect (Morais Júnior et al. 2018b; Baertschi et al. 2021).

The collaborative upland rice breeding program between CIAT (International Center for Tropical Agriculture, member of the CGIAR centers, now known as the Alliance Bioversity-CIAT) and Cirad (French Agricultural Research Centre for International Development) has developed synthetic populations managed through RS. The orientation towards population improvement took place in the 1990s following the observation of the declining crop genetic diversity among improved rice germplasm (Martinez et al. 2014). An RS scheme consists of three main steps conducted recurrently. It is summarized as follows: i) evaluation of a sub-set of families, ii) selection of the best ones based on progeny mean performance, iii) inter-crossing of the selected families to develop the next cycle of selection. In the Cirad-CIAT program, the RS scheme applied to the autogamous rice was facilitated through the use of a recessive nuclear male-sterility gene (ms-IR36, reviewed in Frouin et al. 2014). The breeding program has two distinct locations in Colombia to develop improved populations and inbred lines and to apply shuttle breeding. This shuttle breeding allows the application of two cycles of selection and generation advance in a year; selecting in the target location for adaptation to local conditions during the main season, while advancing generation in a favorable location during the off-season, selecting for traits less impacted by the environment. While one location is the target production environment for the upland rice, often subjected to abiotic and biotic constraints, the second location benefits from favorable conditions throughout the year with limited pathogen pressure. The basis for the current study for optimizing the CIAT-Cirad upland rice breeding scheme is a proof-of-concept that GS is feasible in the context of RS shuttle breeding with two contrasting locations for phenotyping. An ideal situation for improving the breeding scheme would also be to predict candidates as early as possible in the RS scheme for population improvement and for variety development.

Our study was designed to evaluate whether we can develop GP models on a reduced fraction of a large population at an earliest generation. The ultimate goal remains to reduce phenotyping effort and to effectively apply GS to select the breeding candidates based on their GEBV (genomic estimated breeding values) in a target production environment. Our objectives are to: i) optimize a calibration model with methods considering different integration of the G × E interaction term and applying sparse testing; ii) apply the GS scheme as early as possible during the breeding steps by using multi-generation phenotyping, and iii) assess whether selection of optimized TS can improve the PA. The potential of the various scenarios to offer efficient and cost-effective methods to apply recurrent GS in our breeding program will be discussed.

## Material and Methods

### Population Development

The training and validation sets were both derived from a rice synthetic population (PCT27) belonging to the tropical japonica group of rice (*Oryza sativa* L.). The population development was described earlier (Grenier et al. 2015; Baertschi et al. 2021). Among the S_0_ fertile plants extracted from the PCT27 population, 384 were used for training the model (PCT27A), while another set of 334 (PCT27B) was considered for validation of the model. All 384 entries of PCT27A were advanced to the S_0:2_ and S_0:3_ and only the PCT27B was advanced to the S_0:4_ generation. All generation advancement was performed by bulk harvesting seeds from 15 to 20 male fertile plants per line per generation. In this work, generation is used to describe the number of selfing steps that were done on a family and does not describe two populations separated by a recombination step. A set of 50 randomly selected families from the S_0:2_ generation extracted from the set considered for model calibration (PCT27A) and designed as "temporal checks" (TC) was included in each trial to account for the year effect within each location. These 50 TC at the S_0:2_ generation were evaluated for three years in each location to assess the year effect without confounding it with the effect of the generation difference of the 334 entries evaluated in the various trials. The 50 TC were preferred to a few inbred lines as they were genetically closer to the families studied in the experiment, representative of the population and thus enabling a better assessment of the year effect under the conditions of the present study.

### Genotyping

Genotyping-by-sequencing was performed on the 718 S_0_ plants as described in Baertschi et al. (2021). Briefly, DNA libraries were prepared at the Regional Genotyping Technology Platform (http://www.gptr-lr-genotypage.com) hosted at Cirad, Montpellier, France and were single-end sequenced in a single-flow cell channel (i.e., 96-plex sequencing) using an Illumina HiSeq2000 (Illumina, Inc.) at the Regional Genotyping Platform (http://get.genotoul.fr/) hosted at INRA, Toulouse, France. The fastq sequences were aligned to the rice reference genome, Os-Nipponbare-Reference-IRGSP-1.0 (Kawahara et al. 2013) using Bowtie2 with the default parameters. Nonaligning sequences and sequences with multiple positions were discarded. Single-nucleotide polymorphism (SNP) calling was performed using the Tassel genotyping-by-sequencing pipeline v5.2.29 (Glaubitz et al. 2014). The filters applied to loci are the missing data (< 20%), the depth for each data point (> 10), the minor allele frequency (> 2.5%) and the bi-allelic status of SNPs. To limit the probability of under-calling a heterozygous site, the read depth for SNP calling was set to a minimum of 10, so that the probability of undercalling a heterozygous site was limited to a theoretical maximum of 0.2% (Swarts et al. 2014). Missing data were imputed using Beagle 4.1 embedded in the R package Synbreed v0.11-22 (Wimmer et al. 2012). The genetic characterization of the two population sets is presented in supplementary Tables and Figures. A total of 713 successfully genotyped S_0_ plants with 9,928 SNP markers distributed among the 12 rice chromosomes (Additional file 1: Fig. S1) were used in this study. The MAF distribution among the 713 S_0_ reflects a population where rare alleles were not depleted, which fits well with the long-term objectives of the breeding program based on population improvement. The degree of allelic fixation varied greatly between the genotypes but remained relatively low for individuals at the S_0_ generation (Additional file 1: Table S1). Considering the rather large average linkage disequilibrium (Additional file 1: Table S2) and the slow linkage disequilibrium decay observed, the average marker density (1 SNP every 40 kb) was considered sufficient to allow the capture of all linked QTLs with the SNP matrix in hand. Globally, the 713 genotypes as two random fractions extracted from a large population did not show any structuring (Additional file 1: Fig. S2).

### Field Trial and Phenotyping

Field phenotyping was performed at two locations in Colombia from 2017 to 2020: the experimental field at CIAT-HQ in Palmira (PAL) located in the Valle del Cauca, Colombia (3.50° N–76.35° W, 1000 masl) and an experimental location in Santa Rosa (SRO) property of the Colombian National Federation of rice growers (Fedearroz), located in the Oriental plains of Colombia, in the department of Meta, Colombia (4.03° N–73.48° W, 300 masl). SRO is within a rice-growing area, where the crop is directly seeded, and cultivated under rainfed conditions during the main cropping season, May to September, with the natural occurrence of various diseases such as blast. Upland rice is commonly grown under rainfed conditions and therefore the SRO location is our target selection site. In the PAL location, however, rice is cultivated with irrigation supply throughout the crop cycle, freeing rice trials from any planting time constraints, and the location is naturally free of known diseases unless purposely exposed to them. PAL is thus a surrogate location.

A total of six field trials were conducted during three growing seasons at the two different locations. Field trials for the S_0:2_, S_0:3_ and S_0:4_ generations were established in PAL on 4 December 2017, 10 December 2018 and 26 December 2019, respectively and in SRO on 12 May 2017, 30 May 2018 and 20 May 2020. PCT27A was phenotyped at the S_0:2_ and S_0:3_ generations, whereas PCT27B was only phenotyped at the S_0:4_ generation. At each location, the experimental design followed a lattice with 8 blocks and three repetitions and included the 334 families and the 50 S_0:2_ TC lines. In PAL, trials were established after transplanting 3-week-old seedlings in a bundled field. The plot size was two rows of 17 plants with 25 cm between plants and between rows. Fertilizer application was split, with NPK nutrients (377 kg/ha urea, 188 kg/ha DAP, 189 kg/ha KCl) added at 25 and 35 days after transplanting. Irrigation was maintained continuously to ensure a 25 cm layer of water in the field until a week prior to the crop maturation period. In SRO, the trials were established by direct seeding of two 4 m-long rows, spaced by 26 cm at a density of 1 g of seed per linear meter. Split fertilizer application was performed according to the recommended application for growing tropical japonica rice in upland soil conditions (230 kg/ha urea, 217 kg/ha DAP, 150 kg/ha KCl). The trial was rainfed and soil property allowed good water drainage and favorable upland conditions. Phytosanitary treatment was applied in SRO to prevent blast outbreaks.

Four traits were measured following the IRRI Standard Evaluation System (IRRI 2013). Flowering date (FL) was expressed as the number of days after crop establishment—being either the date after transplantation (PAL) or sowing (SRO)—when 50% of the plants within a plot reached anthesis. Plant height (PH) was calculated as the average height measured in centimeters of five plants with their panicle extended. Grain yield (YLD) was obtained by weighing the grains collected within each plot after discarding the plants at the start and end of each plot. For each harvested plot, percent humidity was measured and used to correct the weight of collected grains, expressed in grams per plot, for a relative humidity of 14%. The YLD value was neither adjusted for the plot size nor for the count of fertile plants. The grain zinc concentration (ZN), expressed in parts per million (ppm), was measured on a subsample of collected grains, previously polished in Teflon equipment, using energy dispersive X-ray fluorescence spectrometry (X-supreme 8000, Oxford Instrument, Shanghai, CN) available at the CIAT-HQ Nutritional Laboratory.

The exact same phenotyping procedure was used for generations S_0:2_, S_0:3_, and S_0:4_. The 50 TC were phenotyped as S_0:2_ in all the trials (without generation advance) allowing measurement of the year effect per location (Additional file 1: Tables S3 and S4).

### Statistical Analyses

#### Descriptive Statistics

The raw data were checked per trial for outliers using the boxplot.stats function of the R package "stats" (R Development Core Team 2018) with a coefficient of 1.5, which means that outliers were identified if the phenotypic values were outside 1.5 times the interquartile range above the upper quartile and below the lower quartile. No outliers were discarded. Variance decomposition was performed using the lmer function of the R package "lme4" (Bates et al. 2015). The following mixed model was used for each trial independently:

1 $${y}_{ijkl}=\mu + {Loc}_{i}+ {{\mathrm{Rep}}_{j}(Loc}_{i})+{Bl}_{k}\left({Rep}_{j}\left({Loc}_{i}\right)\right)+{g}_{l}+ {g}_{l}({Loc}_{i})+ {e}_{ijkl}$$

where ${y}_{ijkl}$ is the vector of phenotypic values, $\mu$ is the overall mean of the phenotypic values, $Loc$ is the fixed effect of the location (PAL or SRO), $Rep$ is the fixed effect of the replicate (from 1 to 3) within a location, $Bl$ is the random effect of the block k (from 1 to 8) nested in a location and a replicate with distribution $Bl\sim N(0,{\sigma }_{Bl}^{2})$, $g$ is the random effect of the genotype (family) with distribution $g\sim N(0,{\sigma }_{g}^{2})$, ${g}_{l}({Loc}_{i})$ is the random nested effect of the genotype within a location, which is the genetic by environment interaction effect, and ${e}_{ijkl}$ is the residual considered as a random effect with distribution $e\sim N(0,{\sigma }_{e}^{2})$. Inter-annual variance and genotype by year interaction variance were only considered for the 50 TC evaluated in each site over three years (Additional file 1: Table S4). For the 334 families of PCT27A evaluated at S_0:2_ in 2017 and S_0:3_ in 2018, the year effect was thus confounded with any potential generation effect.

Broad sense heritability (H^2^) was estimated using the following equation:

2 $${H}^{2}=\frac{{\sigma }_{g}^{2}}{{\sigma }_{g}^{2}+\frac{{\sigma }_{g(Loc)}^{2}}{NE}+\frac{{\sigma }_{e}^{2}}{NR}}$$

where ${\sigma }_{g}^{2}$ is the variance associated with genotypes, ${\sigma }_{g:loc}^{2}$ is the genetic by environment interaction effect variance, ${\sigma }_{e}^{2}$ is the residual variance, NE is the harmonic mean of the number of locations per genotype and NR is the harmonic mean of the number of replicates per genotype across the two locations.

To estimate correlations between environments, for each trait, correlations of phenotypic values between the two locations were performed using the rcorr function of the R package "Hmisc" (Harrell 2021).

#### Genomic Prediction

Genomic prediction models were developed as a two-stage method. First, to correct for the fixed effects of location, replicate and bloc, best linear unbiased estimations (BLUE) were estimated for each trait within the location using the lmer function and the following model:

3 $${y}_{jkl}=\mu +{Rep}_{j}+{Bl}_{k}\left({Rep}_{j}\right)+{g}_{l}+ {e}_{jkl}$$

where ${g}_{l}$ is the fixed effect of the genotype l.

The GP model was run by generation and the BLUE values for each trait were used to compare with the predictions.

**Fig. 1:**
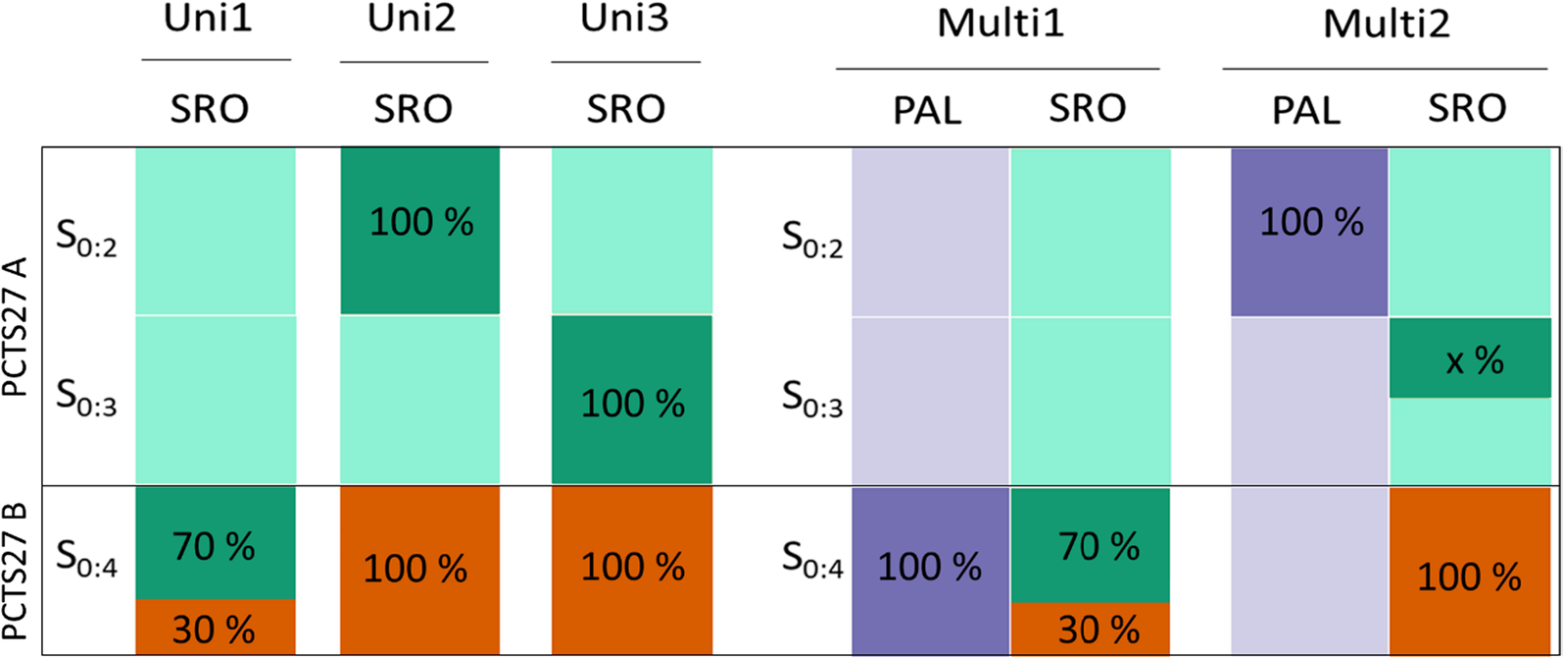
The different scenarios of calibration and validation of the GP models used to predict the phenotype. Among the fertile plants extracted from the PCT27 population, 384 were used to train the model (PCT27A), while another set of 334 (PCT27B) was considered for validation of the model. The different scenarios of calibration and validation of the GP models used to predict the phenotype of the PCT27B at the S_0:4_ generation in Santa Rosa (SRO). The red area represents the validation set (VS), the green and blue represent the training set (TS), from Santa Rosa and Palmira (PAL), respectively. The percentage in the colored areas represents the fraction of the population used to calibrate or validate the model. The x% of S_0:3_ families phenotyped in SRO included in the TS in Multi2 scenario varied from 25, 50 and 75%. Scenarios can be summarized as follow: Uni1: cross-validation to estimate the predictive ability of a model calibrated with the information of PCT27B in a single location (SRO); Uni2 and Uni3: families from PCT27A at generation S_0:2_ or S_0:3_, respectively, were used as a TS to estimate the genomic breeding values of all the families of PCT27B at generation S_0:4_. Only one environment (SRO) was included in these scenarios. Multi1: data from two locations (PAL and SRO) from a single generation (S_0:4_) were used, TS was composed of 100% and 70% of the PCT27B families phenotyped at PAL and SRO, respectively, and the VS was composed of the remaining 30% of the PCT27B families phenotyped in SRO. Multi2: TS consisted of 100% of the families phenotyped in PAL at the S_0:2_ generation and 25, 50 or 75% of the families measured at SRO at the S_0:3_ generation

Genomic predictions were performed under several scenarios depending on the families included in the TS and the VS, as illustrated in Fig. 1:

1. The first scenario (Uni1) was a CV to estimate the PA of a model calibrated with the information of PCT27B in a single location (SRO). The genotypes of plants at the S_0_ generation and the phenotypes of their derived progenies at the S_0:4_ generation were used to predict the values of S_0:4_ families in SRO. In this scenario, the TS consisted of a random draw of 70% of PCT27B and the remaining 30% constituted the VS.
2. The second scenario (Uni2) was used to evaluate the suitability of the models when families from PCT27A at generation S_0:2_ were used as a TS to estimate the genomic breeding values of all the families of PCT27B at generation S_0:4_. Only one environment (SRO) was included in this scenario.
3. The third scenario (Uni3) was similar to Uni2, except the calibration was performed with PCT27A families at generation S_0:3_.
4. The fourth scenario (Multi1) was performed to highlight the impact of G × E interactions. Data from two locations (PAL and SRO) from a single generation (S_0:4_) were used. The TS was composed of 100% and 70% of the PCT27B families phenotyped at PAL and SRO, respectively, and the VS was composed of the remaining 30% of the PCT27B families phenotyped in SRO. The 30% of the PCT27B families phenotyped in SRO used for the CV (for which the phenotypic data were removed) were picked by random draw.
5. The last scenario (Multi2) tested the potential of GP using data from PCT27A at generations S_0:2_ and S_0:3_ phenotyped in PAL and SRO, respectively, to predict PCT27B at generation S_0:4_. In this scenario, the calibration was performed on the PCT27A population. The TS consisted of 100% of the families phenotyped in PAL at the S_0:2_ generation and 25, 50 or 75% of the families measured at SRO at the S_0:3_ generation. The choice of the S_0:3_ included in the TS was either randomly drawn or selected through an optimization process, as presented below. The validation was made, as before, with the phenotypes of the whole population PCT27B at the S_0:4_ generation grown at SRO.

Bayesian GBLUP was performed for all the analyses. For the Uni1, Uni2 and Uni3 scenarios, the GP were run using a univariate single-environment model considering only the main genotypic effects using the BGGE package (Granato et al. 2018).

In the Multi1 and Multi2 scenarios, an environment or a G × E interaction random effect was added to the predictive model. To do so, G × E genomic variance matrices were constructed and GP was performed using a Bayesian linear mixed model. Three different multi-environment models were used in the present study all of which are available in the BGGE package (Granato et al. 2018):

(i) A multi-environment model (MM) assuming that genetic effects across the environment are constant, and therefore the absence of G × E. In this model, a single matrix containing the genomic relationships was constructed for the main across-environment effects:
  4 $${y}_{ij}=\mu + {Loc}_{i}+{g}_{j}+{e}_{ij}$$

for ${Loc}_{i}$ and ${g}_{j}$ as described in model (3), with ${g}_{j}$ having a variance–covariance structure following ${g}_{j}\sim N(0,{\sigma }_{g}^{2}G)$, G being the genomic relationship matrix from VanRaden (2008);

(ii) A multi-environment model (MDs), which is an extension of the MM model (4) including a single random deviation effect of the G × E, the G × E effects following the normal distribution ${g}_{j}({Loc}_{i})\sim N(0, {\sigma }_{GxE}^{2}$ G);
  5 $${y}_{ij}=\mu + {Loc}_{i}+{g}_{j}+{g}_{j}({Loc}_{i})+{e}_{ij}$$

(iii) A multi-environment model (MDe) with an environment-specific variance deviation effect for the G × E. The model was the same as for MDs but used a more complex variance–covariance structure for the G × E effects: $${g}_{j}({Loc}_{i})\sim N\left(0,\left[\begin{array}{cc}{\sigma }_{PAL}^{2}G& 0\\ 0& {\sigma }_{SRO}^{2}G\end{array}\right]\right)$$ ${\sigma }_{PAL}^{2}$ and ${\sigma }_{SRO}^{2}$ being environment-specific variances and $G$ the genomic relationship matrix. Full details about these models can be found in Granato et al. (2018). All GPs were performed using the R package BGGE (Granato et al. 2018) with the following parameters: burn-in = 2,000, nIter = 70,000 and thin = 100.

#### Training Set Optimization

Considering the Multi2 scenario including multi-generations and two environments, one of the objectives was to test whether it was possible to reduce the phenotyping effort in SRO in generation S_0:3_. In this scenario, the CDmean criterion, based on GBLUP, was used to select the TS and compared to randomly selected TS. This choice of the CDmean sampling algorithm to optimize the TS was made due to the fact that this method, minimizing the relationship between genotypes in the TS and maximizing the relationship between TS and VS, is relevant for long-term selection (Isidro et al. 2015). Twenty-five percent, 50% or 75% of the S_0:3_ phenotyped individuals grown in SRO were included in the TS. Rincent et al. (2012) proposed this optimization criterion based on the expected reliability of contrast predictions and defined as the squared correlation between true and predicted contrasts of genetic values. The parameters used were similar to those used for the previous model, adding a value of 1 for the variance ratio λ (with λ = (1 − h^2^)/h^2^) corresponding to a heritability of 0.5. The R package *TrainSel*, which implements the genetic algorithm for the optimization of the TS selection, was used in this study (Akdemir et al. 2021a, b). The parameters for the genetic algorithm were set as follows: number of iterations 200, population size 300, and number of elite solutions at each iteration 10. The optimization was repeated for each scenario.

#### Model and Scenario Comparison

For each model and scenario, the PA was computed as the correlation between predictions and the BLUE adjusted by trial. To ensure that variations in accuracy between models and scenarios were not due to stochastic effects, all predictions (except for Uni2 and Uni3 for which no stochastic effect was estimable) were replicated 100 times, allowing the mean and standard deviation of each model to be estimated and compared using all the predictive abilities. The comparison of the prediction models was performed with a simple linear model considering the scenario as fixed effect and after Fisher Z statistics [Z = 0.5*log((1 + PA)/(1 − PA))] of the PA data as in Ben Hassen et al. (2018b).

### Economic Estimation of the Cost of Strategies

To compare the various strategies, cost estimates were obtained considering the types of trials, their size and location, and whether in PAL or SRO. The trials were defined as “generation advance” or “phenotype evaluation”. While the trial for generation advance was small and relatively simple in management, with only two 3 m-long rows carried out in PAL and major labor activity at sowing, transplanting and harvesting, the experimental set up for phenotype evaluation included a repeated design and additional labor forces for crop management and phenotyping. A unit cost (1X$) for the phenotype evaluation of 400 genotypes (1200 plots) was defined for each location (1X$_PAL_ and 1X$_SRO_) according to the field management, labor, inputs, and transportation cost. The cost for the generation advance trial, which was conducted only in PAL, was estimated to be 40% of the cost of the phenotyping experiments (0.4X$_PAL_). This reduced cost was assessed by considering a smaller field size that did not require experimental design or repetition, with fewer field activities as no phenotyping was conducted. In the end, seed multiplication is significantly cheaper by reduced field management, input and labor cost. The final cost estimates for all the scenarios were then compared.

## Results

### Phenotypic Performances

For all four traits measured on the PCT27B, differences were observed between the two locations (Table 1). On average, FL was 6 days earlier and plant height (PH) 22 cm shorter at SRO than at PAL. YLD was greatly reduced (5.5 times lower) at SRO, and ZN was 12.6 ppm higher at SRO than at PAL. Coefficients of variation of all traits were higher at SRO than at PAL. Phenotypic correlations between locations were relatively low, and ranged from 0.216 (for YLD) to 0.319 (for FL).

**Table 1:** Descriptive statistics for the PCT27B phenotyped at the S_0:4_ generation in two locations; Palmira (PAL) and Santa Rosa (SRO) with mean, standard deviation (SD), min, max, coefficient of variation and the phenotypic correlation (Pearson) between locations (*p*-values < 0.0001)

Trait^1^	Location	PCT27B S_0:4_ generation					
		Mean	SD	Min	Max	Coefficient of variation	Corr
FL	PAL	87.38	3.84	78	96	4.39	0.319
	SRO	81.68	5.73	69	96	7.02	
PH	PAL	120.4	4.98	113.2	128.2	4.14	0.229
	SRO	97.84	8.89	75	121	9.09	
YLD	PAL	759.6	184.1	304.6	1240.1	24.2	0.216
	SRO	137.0	50.1	65.4	270.5	36.6	
ZN	PAL	14.68	1.83	10	19.6	12.5	0.313
	SRO	27.27	3.65	18	37.5	13.4	

^1^Traits are flowering date (FL), plant height (PH), grain yield per plot (YLD) and grain zinc concentration (ZN)

For each trait measured, an analysis of variance components was performed (Table 2 and Additional file 1: Table S6). Surprisingly, the proportion of variance explained by the genotype effect was particularly low for PH, explaining the near-zero H^2^. However, distinguishing the two locations, it appeared that H^2^ was close to zero for PH measured at PAL, which might potentially be explained by experimental bias. However, data from PAL in S_0:4_ was not considered except in the Multi1 scenario, and H^2^ was high for PH measured at SRO (H^2^ = 0.70), which is the phenotype in the target site used to obtain the PA. For all other trait combinations, H^2^ was moderate, ranging from 0.20 to 0.87, with a lower H^2^ for YLD than for FL and ZN when both locations were included. The G × E effect accounted for a large part of the variance with an explained proportion ranging from 30.9 to 36.9% for the four traits.

**Table 2:** Variance decomposition and broad sense heritability (H^2^) by trait for the PCT27B at S_0:4_ generation with both locations or within each location (PAL and SRO)

Trait	Variance component	PCT27B S_0:4_—All		PCT27B—PAL		PCT27B—SRO	
		Proportion of variance	H^2^	Proportion of variance	H^2^	Proportion of variance	H^2^
FL	Genotype	24.7	0.51	47.7	0.78	67.2	0.87
	Location:Genotype	36.1		–		–	
	Bloc:Rep:Location	6.20		–		–	
	Bloc:Rep	–		12.1		3.80	
	Residuals	33.0		40.0		28.8	
PH	Genotype	0.60	0.02	0	0.00	41.3	0.70
	Location:Genotype	36.9		–		–	
	Bloc:Rep:Location	4.20		–		–	
	Bloc:Rep	–		10.1		4.90	
	Residuals	58.4		89.8		53.6	
YLD	Genotype	6.80	0.2	44.1	0.72	38.8	0.68
	Location:Genotype	35.4		–		–	
	Bloc:Rep:Location	2.80		–		–	
	Bloc:Rep	–		5.40		5.6	
	Residuals	55		50.3		55.4	
ZN	Genotype	23.4	0.51	48.8	0.81	53.9	0.80
	Location:Genotype	30.9		–		–	
	Bloc:Rep:Location	5.60		–		–	
	Bloc:Rep	–		15.8		6.10	
	Residuals	40.1		35.3		39.8	

### Single Location Calibrations

The potential of GP was first tested for the prediction of phenotypes in the target location, i.e., in SRO (Table 3). Cross-validation (CV) was used both within PCT27B (Uni1) and across sub-populations with PCT27B and PCT27A (Uni2 and Uni3). For prediction within PCT27B on progenies at the S_0:4_ generation (Uni1), PA ranged from 0.19 for ZN to 0.30 for PH and YLD. The PA using models calibrated on the PCT27A (S_0:2_ progenies for Uni2 or S_0:3_ progenies for Uni3) were higher than Uni1 only for FL and ZN. Increase in PA was significant for FL in Uni2 (PA = 0.32) or Uni3 (PA = 0.30) compared to Uni1 (PA = 0.25 ± 0.08). The difference was even greater for ZN, regardless of the generation used (Uni2 or Uni3), with PA around 0.30 compared to Uni1 (PA = 0.19 ± 0.08). No difference in PA was observed for PH among all Uni scenarios. For YLD, the PA were lower when using the two sub-populations, but the difference was only significant in the case of Uni3 (PA = 0.24) compared to Uni1 (PA = 0.30 ± 0.08).

**Table 3:** Predictive ability (PA, LSmeans ± standard deviation or LSmeans) for the three "Uni location" scenario combining different make-up of the training set and validation set

Training set	Validation set	Scenario	FL	PH	YLD	ZN
PCT27B S_0:4_ (70%)	PCT27B S_0:4_ (30%)	Uni1	0.25 ± 0.08	0.30 ± 0.07	0.30 ± 0.08	0.19 ± 0.08
PCT27A S_0:2_ (100%)	PCT27B S_0:4_ (100%)	Uni2	0.32	0.31	0.27	0.31
PCT27A S_0:3_ (100%)	PCT27B S_0:4_ (100%)	Uni3	0.30	0.29	0.24	0.29

**Fig. 2:**
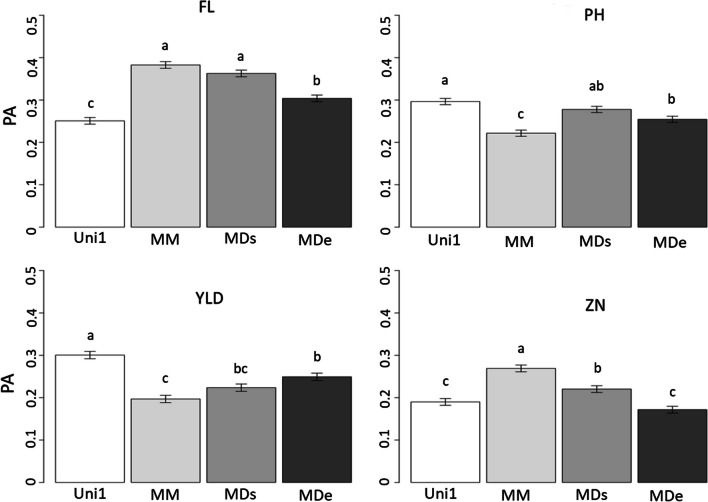
Predictive ability (LSmeans with error-bars representing the standard error) within the Uni1 and Multi1 scenarios with three different models. We used the multi-site model without genotype by environment interaction (MM) and the multi-site model including the genotype-by-environment interaction with similar variances between environments (MDs) or with different variances between environments (MDe). Calibration and validation were performed within the PCT27B population phenotyped at the S_0:4_ generation in Santa Rosa (SRO) for the four traits of interest: flowering date (FL), plant height (PH), grain yield per plot (YLD) and grain zinc concentration (ZN). TS included 100% of the records in Palmira (PAL) and 70% of the records in SRO for all the models except SM where the TS included only 70% of the phenotypes recorded in SRO. For all models VS was 30% of phenotypes in SRO. Within a trait, the letters represent significant differences between models (*p*-value < 0.05)

The description of the scenarios is in Fig. 2

### Genomic Prediction and G × E Interactions

The Multi1 scenario considered one generation but two locations. It was tested to assess the utility of including the G × E interaction in the GP models. Using this scenario, it was possible to estimate the PA of models including both locations with a fixed location effect (MM), and the G × E interaction effect with single or two different variances for each of the two locations (MDs and MDe, respectively). The PA obtained with the Multi1 scenario and the three models are shown in Fig. 2 and compared with the Uni1 scenario. From these analyses, it appeared that only for FL and ZN, the PA using multi-location calibration resulted in significantly higher PA than with model Uni1 with maximum PA increase of + 0.13 and + 0.08 for FL and ZN, respectively. These two traits responded in broadly the same way: PA using the MM model had the highest values, followed by MDs and MDe. Only for FL were all three multi-location models greater than Uni1, while for ZN the MDe gave a PA similar to that with Uni1. In the case of PH and YLD, the PA from multi-location models did not improve the PA compared to the Uni1 model.

### Multi-Generation and Multi-Environment Genomic Prediction

**Fig. 3:**
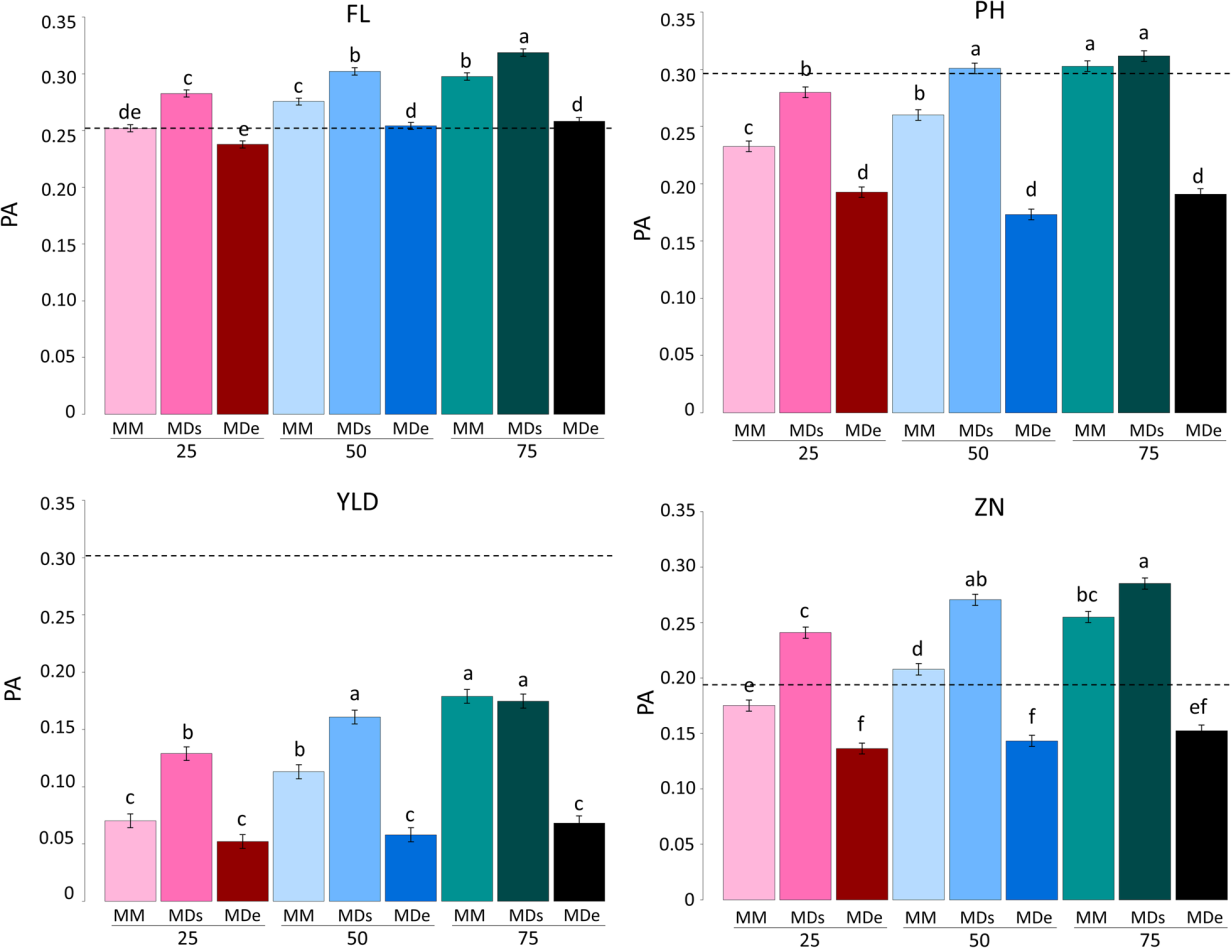
Predictive ability (LSmeans with error-bars representing the standard error) for the multi-site model (Multi2 scenario) without G × E interaction (MM, lighter color), including the genotype-by-environment interaction with similar variances between environments (MDs) or with different variances between environments (MDe, darker color). The training set was constituted with the phenotypes of the PCT27A at generation S_0:2_ in Palmira (PAL) at and a proportion (25, 50 or 75%) chosen randomly of S_0:3_ in Santa Rosa (SRO). Validation was performed with the phenotypes of the PCT27B at generation S_0:4_ in Santa Rosa (SRO) for the four traits of interest: flowering date (FL), plant height (PH), grain yield per plot (YLD) and grain zinc concentration (ZN). Dashed horizontal lines represented the PA of the Uni1 scenario. Within a trait, the letters represent significant differences between models (*p*-value < 0.05)

Our objective was to combine approaches of early-generation prediction using a TS phenotyped in S_0:2_ and S_0:3_ and multi-environment GP, as presented in the Multi2 scenario (Fig. 3). Globally, the same tendencies were drawn for all the traits as the more phenotypes of S_0:3_ families phenotyped in SRO included in the TS, the higher the PA. The greatest PA were achieved with 75% of S_0:3_ families included in the TS with PA = 0.32, 0.31, 0.18 and 0.29 for FL, PH, YLD and ZN, respectively. Regardless of the TS size, the PA of the MDs model were usually the highest or comparable to the MM model, although these differences diminished with the increase of the TS size (75% of the S_0:3_ phenotypes). PA obtained with the MDe models were low for all traits and varied only slightly with TS size. For all traits except YLD, the best PA were obtained with a TS size of 75% and the MDs model. Only for PH and ZN did decreasing size to 50% not significantly reduce PA. For YLD, the MM and MDs models including 75% of S_0:3_ families and MM including 50% of S_0:3_ families were the best models, although far from the values of Uni1.

### Optimization of the Training Set

**Fig. 4:**
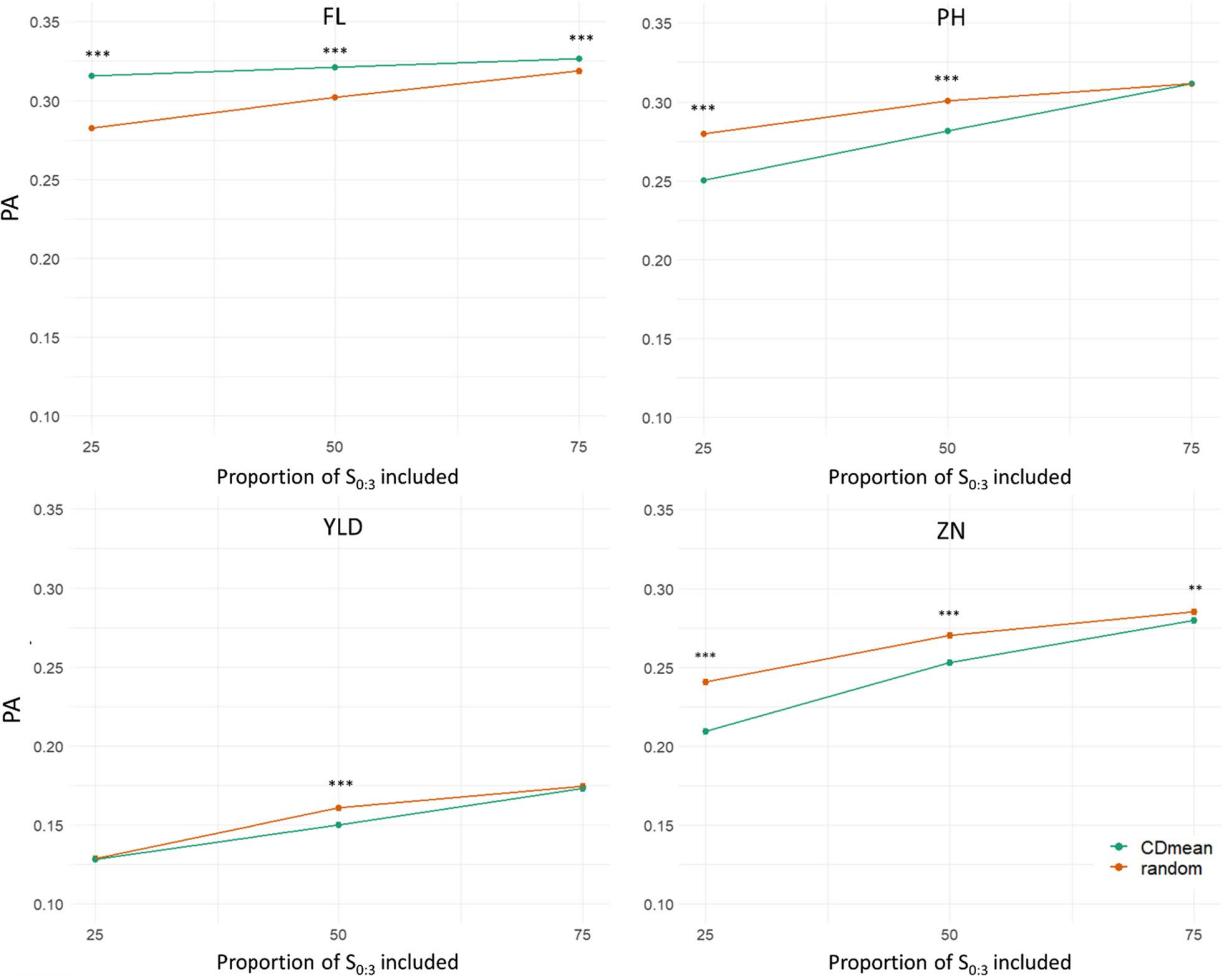
Predictive ability (LSmeans with error-bars representing the standard error) for the multi-site model (Multi2 scenario) including the genotype-by-environment interaction with similar variances between environments (MDs). The training set was performed with the phenotypes of the PCT27A at generation S_0:2_ in Palmira (PAL) and a proportion (25, 50 or 75%) chosen randomly (in orange) or using the CDmean optimization method (in green) of S_0:3_ in Santa Rosa (SRO). Validation was performed with the phenotypes of the PCT27B at generation S_0:4_ in Santa Rosa (SRO) for the four traits of interest: flowering date (FL), plant height (PH), grain yield per plot (YLD) and grain zinc concentration (ZN). Within a proportion of S_0:3_ in Santa Rosa (SRO) included in the TS, the asterisks represent significant differences between models (*p*-value < 0.05)

Within the Multi2 scenario, one way to gain PA while keeping the phenotyping effort low would be to optimize the choice of individuals to be included in the TS. As TS optimization method, CDmean was performed to best choose the S_0:3_ families phenotyped at SRO to be included in the TS. The full comparison of the sampling methods across the three TS sizes and the three G × E models is shown in the supplementary file (Additional file 1: Table S7). To simplify understanding, we chose to present the effect of the TS selection only with the MDs model across the three TS sizes (Fig. 4). Optimizing the selection of S_0:3_ families phenotyped in SRO to be included in the TS increased the PA only for FL (from + 0.008 to + 0.034 according to the proportion of S_0:3_ included in the TS) compared to a random draw of the TS. For PH and ZN, the sampling based on CDmean resulted in significantly lower PA with an inclusion of 25% (loss in PA from 0.280 to 0.250 and from 0.240 to 0.210 for PH and ZN, respectively) and 50% (reduction of PA from 0.301 to 0.282 and from 0.270 to 0.253 for PH and ZN, respectively) of S_0:3_ in the TS. Reduction in PA was also detected with the CDmean selection for including 50% of S_0:3_ in the TS in the case of YLD (loss PA from 0.161 to 0.150). For these last three traits, an increase of PA with TS size increase was visible whatever the model used. For YLD, the selection of the S_0:3_ families phenotyped at SRO included in the TS had no impact on PA for 25 and 75% of S_0:3_ included in the TS. For this trait, the increase in PA with TS size had the same tendency as with the random sampling.

### Economic Estimates for the Various Scenario

We compared our five scenarios in terms of time spent for the calibration and relative cost considering the generation advance and phenotype evaluation trials. We considered the activities further than just the calibration of a GP model and included the preparation of the germplasm for the activity of elite line development which was set to start at the S_0:4_ generation. While the GP model could be built in 1.5 years for the Uni2, Uni3 and the Multi2 scenarios, it took 2.5 years for the Uni1 and Multi1 scenarios (Table 4). The calibration based on phenotype obtained at a more advanced generation (S_0:4_ progenies), Uni1 and Multi1, resulted in higher cost due to the need for multiplication steps. The GP using the phenotypes gathered from two locations to predict the performance in one target selection location was more costly, as two trials for phenotype evaluation were required (Multi versus Uni scenarios). However, defining an optimal calibration set with reduced TS in one of the two locations resulted in a significant reduction in cost (Multi 2 with 1.4X$_PAL_ + 0.6X$_SRO_ versus Multi 1 2.2X$_PAL_ + 1X$_SRO_). Capitalizing on efforts in a given field trial for both generation advance and phenotype evaluation yielded the best benefits in terms of time and cost. Furthermore, the Multi 2 scenario allowed us to use the off-season semester in PAL with optimal conditions to produce quality seeds of the whole population and set the phenotyping during the main growing season with only a reduced fraction of the population.

**Table 4:** Time and cost for each scenario to generate the material (generation advance) and phenotype the training set (TS) to calibrate a genomic prediction (GP) model and produce the generation on which to start the pedigree breeding scheme

Scenario	Season*	Generation¶	TS size	Generation advance in PAL	Phenotyping		GP	Total cost
					PAL	SRO		
Uni1	Yr1-A	S_0:1_→S_0:2_	100%	0.4X$_PAL_				1.2X$_PAL_ + 1X$_SRO_
	Yr1-B	S_0:2_→S_0:3_	100%	0.4X$_PAL_				
	Yr2-A	S_0:3_→S_0:4_	100%	0.4X$_PAL_				
	Yr2-B	No activity						
	Yr3-A	S_0:4_→S_0:5_	100%			1X$_SRO_	GP	
Uni2	Yr1-A	S_0:1_→S_0:2_	100%	0.4X$_PAL_				0.9X$_PAL_ + 1X$_SRO_
	Yr1-B	S_0:2_→S_0:3_	100%	0.4X$_PAL_				
	Yr2-A	S_0:2_→S_0:3_	100%			1X$_SRO_	GP	
	Yr2-B	S_0:3_→S_0:4_	Sel. fam	0.05X$_PAL_				
Uni3	Yr1-A	S_0:1_→S_0:2_	100%	0.4X$_PAL_				0.8X$_PAL_ + 1X$_SRO_
	Yr1-B	S_0:2_→S_0:3_	100%	0.4X$_PAL_				
	Yr2-A	S_0:3_→S_0:4_	100%			1X$_SRO_	GP	
Multi1	Yr1-A	S_0:1_→S_0:2_	100%	0.4X$_PAL_				2.2X$_PAL_ + 1X$_SRO_
	Yr1-B	S_0:2_→S_0:3_	100%	0.4X$_PAL_				
	Yr2-A	S_0:3_→S_0:4_	100%	0.4X$_PAL_				
	Yr2-B	S_0:4_→S_0:5_	100%		1X$_PAL_			
	Yr3-A	S_0:4_→S_0:5_	100%			1X$_SRO_	GP	
Multi2	Yr1-A	S_0:1_→S_0:2_	100%	0.4X$_PAL_				1.4X$_PAL_ + 0.6X$_SRO_
	Yr1-B	S_0:2_→S_0:3_	100%		1X$_PAL_			
	Yr2-A	S_0:3_→S_0:4_	50%			0.6X$_SRO_	GP	

Cost X$_PAL_ and X$_SRO_ are unit prices for the phenotyping of 1200 plots in Palmira (PAL) and Santa Rosa (SRO), respectively

^*^year-semester; ¶ generation planted→generation harvested

## Discussion

Marker-assisted breeding has been advocated as a major player to develop climate-smart and nutrient-dense crop cultivars in a cost- and time-efficient manner (He and Li 2020; Varshney et al. 2021). Adaptation to climatic constraints or enhancing grain quality traits such as grain mineral concentration are often hard to improve using a few target markers to follow key genomic regions (Dias et al. 2018, 2020; Joukhadar et al. 2021). GS, on the other hand, has proven valuable for improving quantitative traits and has had a significant impact in terms of improving genetic gain in plant breeding programs (Bernardo and Yu 2007; Heffner et al. 2011; Rutkoski et al. 2012; Sorrells 2015). Both national and international breeding institutions are embarking on systematic use of molecular markers to improve the efficiency of their programs through the use of genomic breeding (Varshney et al. 2021). Despite efforts to limit labor-intensive phenotyping through mechanization and high-throughput phenotyping, the cost of phenotyping is still high (Bagchi et al. 2016; Rutkoski et al. 2016; Leng et al. 2017; Jimenez et al. 2019). Yet, well-performing GP models rely on quality phenotypes. Thus, even in the context of GS, it remains important to find a way to reduce phenotyping efforts without reducing the PA of prediction models. In addition to high quality in phenotyping, constitution of the TS to calibrate the prediction models has also been shown to strongly influence the PA values (Spindel et al. 2015; Berro et al. 2019; Merrick et al. 2022). The overall objective of the present study was to assess whether GP could efficiently improve the RS scheme in the current Cirad-CIAT program. Specifically, we wanted to investigate which TS and which GP models based on the infrastructure of the program would allow the best compromise between PA and cost for the breeding program.

### Genomic Prediction in Recurrent Selection

With a single-environment model, the PA obtained through CV to predict the S_0:4_ families at SRO were relatively low compared to those previously estimated in the literature (reviewed by Bartholomé et al. 2022). In comparison with populations of similar make-up, the PA obtained in the current study for PH (0.30) was below those reported in the populations of 343 S_2:4_ and 174 S_1:3_ where the PA were above 0.50 (Grenier et al. 2015; Morais Júnior et al. 2018a). Although these studies also considered progenies extracted from populations derived from multiparental crosses, they differed by their genetic composition, generation of phenotyping and genotyping, size, effective population size and showed different distributions of variances for the considered traits. All of these factors can impact PA to some degree. Interestingly, for FL the PA were roughly similar between these studies (between 0.23 and 0.26) and all had a relatively high and similar broad sense heritability (H^2^ from 0.51 to 0.87). For YLD, the values of PA obtained with the S_0:4_ TS were lower (0.30) than in S_1:3_ of Morais Júnior et al. (2018a) (0.44) and higher than in the S_2:4_ (0.27). These differences could be partly due to the degree of repeatability in the case of S_1:3_ (H^2^ = 0.54) being higher than in any of the other studies (H^2^ below 0.30). Compared to the PA from the CV of Baertschi et al. (2021) on the S_0:2_ and S_0:3_ generations of the PCT27A, the PA obtained through CV in our set of S_0:4_ derived from the PCT27B were lower for most traits except for YLD where the estimates were similar (PA = 0.30 for S_0:4_ versus PA = 0.26 and 0.35 for S_0:2_ and S_0:3_, respectively) (Additional file 1: Table S7). Although the traits of interest in our study ranged from oligogenic to polygenic, as in other rice GP studies (Spindel et al. 2015; Ben Hassen et al. 2018a), a potential relation between the genetic architecture of the traits and the PA is not evident.

Although PA were relatively moderate (0.19–0.30), GS still represents a significant advance over the RS breeding scheme. One of the great potentials of GS lies in its ability to increase the selection intensity (Heffner et al. 2009; Hunt et al. 2018; Cobb et al. 2019; R2D2 Consortium et al. 2021). Any increase in selection intensity can positively impact the breeders' equation. Yet, there is still room for improving genetic gain, notably in terms of speed of the breeding cycle. In our rice breeding program, two phenotyping locations are available, one being a location where rice can be grown all year around, which raises the question of whether shuttle breeding and sparse phenotyping could be applied to reduce the breeding cycle length.

### Sparse Testing Approach in Recurrent Genomic Selection

While SRO is the target selection location, it is far away from CIAT-HQ, and more complex to manage within the research activities. PAL is located at CIAT-HQ and is a more practical location for conducting field trials for generation advance and phenotype evaluation. Our objective was thus to concentrate the phenotyping efforts on the surrogate location while keeping relevance for the target environment. In general, across traits, the phenotypic correlations between the two locations were low for the S_0:4_, and lower than those reported in the earlier generation except for YLD (Baertschi et al. 2021). This low phenotypic correlation can easily be explained by the differences in the geography and cultivation practices between PAL and SRO, the former being an irrigated system at 1000 masl, the latter being under rainfed conditions at 300 masl. Both YLD and FL are sensitive to these factors. This low phenotypic correlation between locations suggests a high genotype-by-environment effect and makes accurate prediction across locations more difficult. When location correlations are low, the possibility of accurately estimating the genomic estimated breeding values of non-phenotyped individuals is low (Hunt et al. 2018) and this was reflected in our results.

Sparse testing in the context of rice was conducted by Morais Júnior et al. (2018b). In their single-step reaction norm models accommodating differentially the relationship of genomic data, environmental covariates and their interaction, similar PA was achieved with all the models for all traits, except FL for which inclusion of the environmental covariate effects significantly improved the PA. In our study, including sparse testing improved the PA for all traits but YLD and this can be explained by the low H^2^ across locations. PA for PH was barely increased with the multi-location model, while for FL and ZN, both with relatively good location correlation or H^2^, the PA were improved with any scenario involving multi-location. This direct link between the power of sparse testing and location correlation was also reported by Ben Hassen et al. (2018a). In their study, multi-location calibration significantly improved the PA as the two environments were highly correlated (0.77) and a relatively low G × E effect (10%) was reported for panicle weight. In our case, the correlations between locations were low and, whichever trait was considered, the model accounting for environment-specific variance deviation effect for G × E (MDe) gave similar (for PH and YLD) or reduced (FL and ZN) PA compared with the model accounting for a single random deviation effect of the G × E (MDs). Consideration of heterogenous residual covariance structure for the MET analysis was more important as the levels of G × E interaction were greater (Mathew et al. 2018). These authors showed that, only in cases with a strong genomic correlation between the environments, the multivariate mixed model yielded better PA than the G × E interaction model. The multi-location model for predicting PH and YLD did not improve the PA compared to the single location and the lack of contribution of data from PAL could come from the low correlation between locations (0.23 and 0.22, respectively).

### Optimization of the Scheme and the Training Set

The Multi 2 scenario was suggested to optimize and accelerate the breeding cycles by taking advantage of two available locations and the generation advance process needed to calibrate the model.

The CV strategy used in our Multi 2 scenario was similar to the CV1 or M1 previously described in the literature (Burgueño et al. 2012; Lopez-Cruz et al. 2015; Ben Hassen et al. 2018a; Bhandari et al. 2019), where the genetic values of individuals have to be predicted based only on their genotypic information without any phenotypic information. Interestingly, these authors showed that the PA using multi-environment models (similar to the MDe model we used) and CV strategy were not improved compared to the single-environment models, whatever the trait under consideration. In the present study, PA were similar for FL or strongly reduced for the other traits when using the Multi 2 scenario with MDe compared to the Uni scenarios based on a single location. Furthermore, the multi-environment model including a single random deviation effect of the G × E (MDs model) appeared to be the one with the highest PA.

Often, GP is used to predict the performances of non-phenotyped entries belonging to different subpopulations (Berro et al. 2019), gene bank collections (Tanaka et al. 2021; Rakotondramanana et al. 2022) or progenies derived from the TS (Ben Hassen et al. 2018a). In the case of recurrent GS, the application of GP can select on the next cycle of the population (Morais Júnior et al. 2018a; Labroo and Rutkoski 2022). A factor of major importance in improving the performance of GS is to ensure the individuals included in the TS are closely related to the subjects in the prediction set (Habier et al. 2010; Clark et al. 2012; Osorio et al. 2021). One way to potentially improve the PA in this Multi2 scenario would be to optimize the choice of S_0:3_ families grown at SRO to be included in the TS. Several studies have demonstrated that using a specific optimization method to choose individuals to be included in the TS could improve PA (Rincent et al. 2012; Akdemir et al. 2015, 2021a; Mangin et al. 2019; Isidro y Sánchez and Akdemir 2021). Improved PA with optimized TS can thus help reduce the phenotyping effort without reducing the power of GP. Optimization of the TS resulted in a higher PA for FL (maximum a + 0.034 for the MDs models and 50% of S_0:3_ in the TS), but it did not significantly impact the PA of PH, YLD and ZN, regardless of the model and proportion of S_0:3_ considered. In agreement with the hypothesis of Ben Hassen et al. (2018b), it can be suggested that beyond a specific threshold of TS size, the inclusion of more genetically close individuals in the VS does not improve the PA. It may be possible to go further and hypothesize that adding more individuals than necessary to the TS would degrade the prediction of the model used. As a consequence, the choice of TS size and the relatedness between TS and VS need to be considered with care in predicting genomic estimated breeding values with the best accuracy (Jannink et al. 2010). The main goals of RS are to increase the frequency of favorable alleles and to maintain genetic variability due to recombination at each cycle of RS (Hallauer and Carena 2012). It can be hypothesized cautiously that, with the population, environments and traits studied in this work, the admixture between families resulting from each cycle of RS was high enough to reduce the genetic structuring between families. Consequently, with the optimization method used, we can assume that the TS cannot be optimized, thus explaining why PA were comparable between the random and optimized TS.

### Economic Impact of the Different Scenarios

The Cirad-CIAT scheme is based on two parts: the RS for population improvement and the pedigree breeding for genetic fixation and selection of candidates for variety release. A strategy opted for in the scheme of variety development is to advance the selected families to a relatively good level of genetic fixation (S_0:4_, for which a theoretical 93.75% homozygosity is found) by bulk harvest to maintain the variability within the family, prior to proceeding to a few generations of pedigree breeding. Early phenotyping evaluation trials conducted in the surrogate location, under favorable conditions and during the off season (e.g. in PAL) to calibrate the model, could also serve to multiply seeds for later generation phenotyping. Then, a reduced fraction of the population could be evaluated during the main season with appropriate field management to capture the GxE.

Our findings reveal that, globally, PA was greater when performing calibration with the Uni2 scenario, compared to Uni1, Uni3 or any Multi scenario. When considering the top 50 best ranked families with Uni2, 26–47% were also found to be best ranked when using the Multi2 scenario, which included only 50% of the population of S_0:3_ at SRO in the TS (Additional file 1: Table S5).

The scope of this paper was not to compare the GS versus phenotype selection as reported in the literature (Gorjanc et al. 2017; R2D2 Consortium et al. 2021; Lubanga et al. 2023), but to compare the strategies making use of shuttle breeding to accelerate the population improvement scheme as well as the identification of the best candidate to be included in the pedigree breeding scheme.

The relative gain in time by applying either the Uni2 or the Multi2 scenario versus the other scenarios is estimated to be one year. The main difference between Multi2 and Uni2 is a reduced investment in the target location (SRO). While a fraction of the population is phenotyped in SRO  for the Multi2 model, the Uni2 model includes phenotyping of the whole set. If phenotyping in the target location is more costly than in the surrogate location, because it involves traveling, application of phytosanitary treatments, or prevention of abiotic stress, then the multi-location (Multi2) strategy can be of interest for cost saving, as only a fraction of the population would be phenotyped in the target location. Furthermore, with two locations, if a problem occurs, we still have a phenotyping record of the population in at least one location.

## Conclusion

Our study revealed that GP based on models calibrated with the S_0:2_ generation holds great potential to predict progenies at later generations. This highlights the potential for early-generation calibration of GP models that phenotype progenies, while still in the segregating generation. Based only on the PA, the best approach depends on the trait considered. A multi-location approach can be similar to or more accurate than a single-location approach considering the added value of the G × E term in the prediction equations as seen for FL, PH and ZN. Furthermore, models integrating multiple locations and generations present a certain advantage as they save time and resources and result in an accelerated breeding cycle. The sparse testing and optimization of the shuttle breeding scheme were evaluated and could thus be considered as a favorable option for conducting the RS breeding program. Finally, although we tested only one method to optimize the TS, the deliberate choice of entries to participate in the calibration did not bring significant improvement to the GP model. The admixture between families and high genetic variability, which are characteristics of populations under RS, holds tremendous promise in increasing selection intensity, provided a very large population of S_0_ plants can be genotyped.

### Supplementary Information

**Additional file 1: Table S1.** Genetic characterization of the two training sets together (genotypes of the 713 S0 plants). **A** Summary information on the distribution, MAF and heterozygosity of the 9 928 SNP loci.** B** Observed heterozygosity (Ho) among the 713 genotypes.** Table S2.** Average linkage disequilibrium (r2) between marker pairs per chromosomes and the distance between markers, considering loci with MAF >2.5%.** Table S3.** Phenotypic correlations between years for the 50 temporal checks repeated in all trials in SRO.** Table S4.** Fixed year effect and variance decomposition for 50 Temporal Checks randomly distributed across the design within each repetition, considering 50 S0:2 lines in the two sites in 2017, 2018 and 2019/2020 trials.** Table S5.** Number of families selected included in the 10, 20 or 50 best ones according to their estimated GEBVs (**A**) in all 24 tested models (Uni1, Uni2, Uni3, 3 models in Multi1 scenario, 18 models in Multi2 scenario), and** B** in the six MDs models of the Multi2 scenario.** Table S6**. Variance decomposition and broad sense heritability (H^²^) obtained using Model 2 by trait and generation.**Table S7.** Predictive ability of the different scenarios and models (means ± standard deviation). For each trait, stars indicate models significantly higher than the Uni1 model.** Figure S1.** Density of SNP markers in the two populations (PCT27A and PCT27B) and the temporal checks set (713 S0 plants) in the 12 chromosomes (chr).** Figure S2.** Biplot from PCA performed on 7766 SNP (after pruning) and 713 S0 plants (PLINK). Grouping by color of PCT27A, PCT27B and the temporal checks (TC belonging to PCT27A).

## Data Availability

All the data used in this study are available in the Cirad dataverse: https://doi.org/10.18167/DVN1/A7DEHI

## Abbreviations

BLUE: Best linear unbiased estimations
CV: Cross-validation
FL: Flowering date
GEBV: Genomic estimated breeding values
GP: Genomic prediction
GS: Genomic selection
G × E: Genotype per environment interaction
MDe: A multi-environment model with an environment-specific variance deviation effect for the G × E
MDs: Multi-environment model including a single random deviation effect of the G × E
MET: Multi-environment trials
MM: Multi-environment model assuming that genetic effects across the environment are constant
PA: Predictive ability
PAL: Palmira location (surrogate location)
PCT27: Name of the rice synthetic population used in the study
PCT27A: Subset of the PCT27 population phenotyped at the S_0:2_ and S_0:3_ generation
PCT27B: Subset of the PCT27 population phenotyped at the S_0:4_ generation
PH: Plant height
RS: Recurrent selection
SNP: Single-nucleotide polymorphism
SRO: Santa Rosa location (target location)
TS: Training set
VS: Validation set
YLD: Grain yield
ZN: Grain zinc concentration
