# Supplementary material for: Optimization of Multi-Generation Multi-location Genomic Prediction Models for Recurrent Genomic Selection in an Upland Rice Population
Source: Rice (N Y). 2023 Sep 27;16:43. doi: 10.1186/s12284-023-00661-0 (PMC10533757; doi:10.1186/s12284-023-00661-0)
Supplement: Supplementary file 1 — Additional file 1: Table S1. Genetic characterization of the two training sets together (genotypes of the 713 S0 plants). A Summary information on the distribution, MAF and heterozygosity of the 9 928 SNP loci. B Observed heterozygosity (Ho) among the 713 genotypes. Table S2. Average linkage disequilibrium (r2) between marker pairs per chromosomes and the distance between markers, considering loci with MAF >2.5%. Table S3. Phenotypic correlations between years for the 50 temporal checks repeated in all trials in SRO. Table S4. Fixed year effect and variance decomposition for 50 Temporal Checks randomly distributed across the design within each repetition, considering 50 S0:2 lines in the two sites in 2017, 2018 and 2019/2020 trials. Table S5. Number of families selected included in the 10, 20 or 50 best ones according to their estimated GEBVs (A) in all 24 tested models (Uni1, Uni2, Uni3, 3 models in Multi1 scenario, 18 models in Multi2 scenario), and B in the six MDs models of the Multi2 scenario. Table S6. Variance decomposition and broad sense heritability (H²) obtained using Model 2 by trait and generation.Table S7. Predictive ability of the different scenarios and models (means ± standard deviation). For each trait, stars indicate models significantly higher than the Uni1 model. Figure S1. Density of SNP markers in the two populations (PCT27A and PCT27B) and the temporal checks set (713 S0 plants) in the 12 chromosomes (chr). Figure S2. Biplot from PCA performed on 7766 SNP (after pruning) and 713 S0 plants (PLINK). Grouping by color of PCT27A, PCT27B and the temporal checks (TC belonging to PCT27A). [file 12284_2023_661_MOESM1_ESM.docx]

Table S1 – Genetic characterization of the two training sets together (genotypes of the 713 S_0_ plants). A) Summary information on the distribution, MAF and heterozygosity of the 9 928 SNP loci. B) Observed heterozygosity (Ho) among the 713 genotypes.


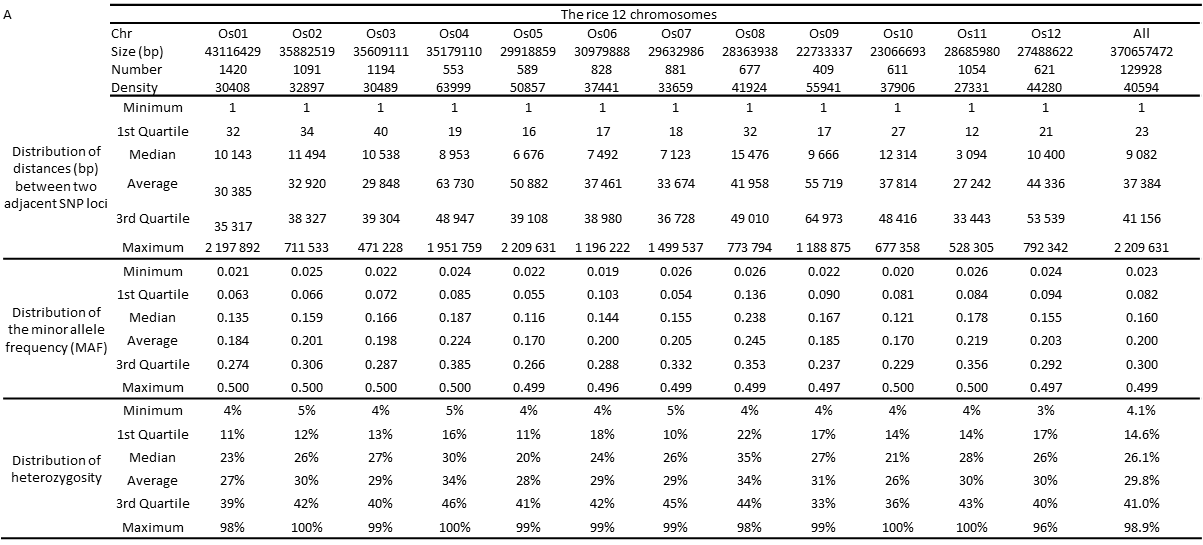


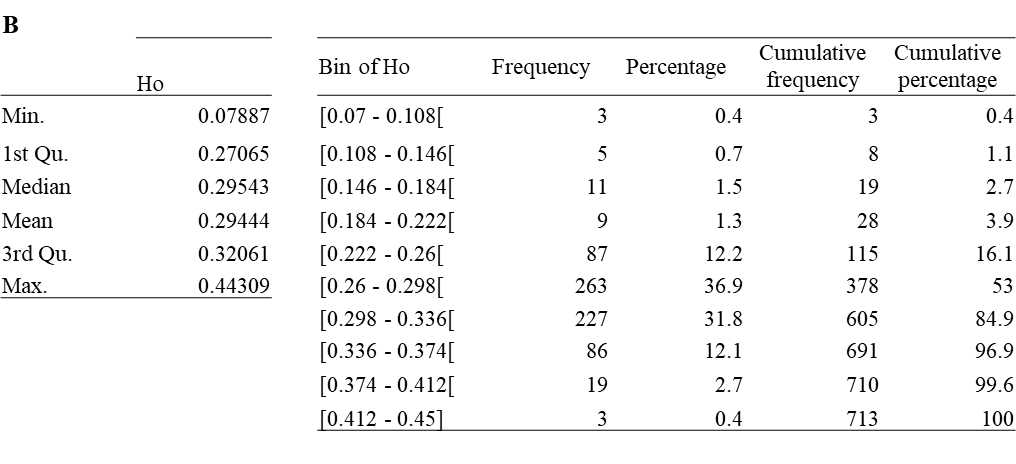


Table S2 – Average linkage disequilibrium (r2) between marker pairs per chromosomes and the distance between markers, considering loci with MAF >2.5%.

|  | The rice 12 chromosomes | | | | | | | | | | | | | |
| --- | --- | --- | --- | --- | --- | --- | --- | --- | --- | --- | --- | --- | --- | --- |
| Distance range (kb) between markers | Os01 | Os02 | Os03 | Os04 | Os05 | Os06 | Os07 | Os08 | Os09 | Os10 | Os11 | Os12 | Average | std |
| ]25:50] | 0.428 | 0.483 | 0.607 | 0.476 | 0.215 | 0.372 | 0.468 | 0.536 | 0.592 | 0.406 | 0.344 | 0.432 | 0.447 | 0.108 |
| ]50:75] | 0.442 | 0.478 | 0.568 | 0.343 | 0.358 | 0.457 | 0.465 | 0.451 | 0.478 | 0.411 | 0.363 | 0.427 | 0.437 | 0.063 |
| ]75:100] | 0.407 | 0.456 | 0.582 | 0.305 | 0.201 | 0.437 | 0.424 | 0.446 | 0.44 | 0.389 | 0.353 | 0.424 | 0.405 | 0.092 |
| ]100:150] | 0.384 | 0.439 | 0.511 | 0.313 | 0.26 | 0.444 | 0.355 | 0.463 | 0.439 | 0.38 | 0.303 | 0.42 | 0.393 | 0.074 |
| ]150:200] | 0.366 | 0.416 | 0.461 | 0.269 | 0.472 | 0.443 | 0.404 | 0.46 | 0.429 | 0.359 | 0.289 | 0.353 | 0.394 | 0.067 |
| ]200:250] | 0.319 | 0.377 | 0.448 | 0.304 | 0.367 | 0.397 | 0.321 | 0.449 | 0.401 | 0.364 | 0.273 | 0.38 | 0.367 | 0.055 |
| ]250:300] | 0.308 | 0.361 | 0.431 | 0.295 | 0.321 | 0.384 | 0.331 | 0.381 | 0.433 | 0.389 | 0.268 | 0.332 | 0.353 | 0.052 |
| ]300:400] | 0.274 | 0.323 | 0.393 | 0.235 | 0.309 | 0.339 | 0.296 | 0.357 | 0.379 | 0.312 | 0.234 | 0.292 | 0.312 | 0.05 |
| ]400:500] | 0.243 | 0.27 | 0.376 | 0.212 | 0.275 | 0.329 | 0.277 | 0.338 | 0.348 | 0.292 | 0.218 | 0.23 | 0.284 | 0.054 |
| ]500:750] | 0.193 | 0.227 | 0.338 | 0.204 | 0.213 | 0.257 | 0.225 | 0.301 | 0.361 | 0.257 | 0.196 | 0.224 | 0.25 | 0.056 |
| ]750:1000] | 0.158 | 0.191 | 0.259 | 0.159 | 0.107 | 0.207 | 0.176 | 0.258 | 0.336 | 0.2 | 0.169 | 0.147 | 0.197 | 0.062 |

Table S3 – Phenotypic correlations between years for the 50 temporal checks repeated in all trials in SRO. Means are in diagonal. FL: Flowering date; PH: plant height; YLD: grain yield; ZN: grain zinc concentration.

| PH | 2017 | 2018 | 2020 |
| --- | --- | --- | --- |
| 2017 | 128.5 | 0.72 | 0.75 |
| 2018 |  | 127.2 | 0.62 |
| 2020 |  |  | 128.4 |

| FL | 2017 | 2018 | 2020 |
| --- | --- | --- | --- |
| 2017 | 86.5 | 0.53 | 0.69 |
| 2018 |  | 88.1 | 0.61 |
| 2020 |  |  | 86.4 |

| ZN | 2017 | 2018 | 2020 |
| --- | --- | --- | --- |
| 2017 | 14.8 | 0.79 | 0.80 |
| 2018 |  | 15.1 | 0.71 |
| 2020 |  |  | 14.9 |

| YLD | 2017 | 2018 | 2020 |
| --- | --- | --- | --- |
| 2017 | 701.7 | 0.64 | 0.52 |
| 2018 |  | 712.8 | 0.57 |
| 2020 |  |  | 715.2 |

Table S4 –Fixed year effect and variance decomposition for 50 Temporal Checks randomly distributed across the design within each repetition, considering 50 S0:2 lines in the two sites in 2017, 2018 and 2019/2020 trials, following the model

y = mu + year + rep:year + bloc:rep:year + genotype + genotype:year + error, underlined effects being considered as random effects.

|  |  | Year effect^a^ | | Variance decomposition^b^ | | |
| --- | --- | --- | --- | --- | --- | --- |
| Trait | Site | range | p-value | G | GxY | (GxY)/total |
| FL | PAL | 84.6-87.71 | ** | 8.70 | 1.60 | 0.08 |
|  | SRO | 79.27-89.01 | *** | 27.00 | 7.10 | 0.15 |
| PH | PAL | 119.4-126.05 | 0.0503 | 31.90 | 1.70 | 0.02 |
|  | SRO | 97.49-117.47 | *** | 15.70 | 4.30 | 0.09 |
| YLD | PAL | 363.7-698.2 | *** | 3816 | 1122 | 0.04 |
|  | SRO | 128.86-399.25 | *** | 1971 | 1074 | 0.16 |
| ZN | PAL | 14.95-15.69 | 0.3920 | 1.584 | 0.10 | 0.03 |
|  | SRO | 22.64-27.78 | *** | 4.25 | 1.22 | 0.13 |

Table S5 – Number of families selected included in the 10, 20 or 50 best ones according to their estimated GEBVs (A) in all 24 tested models (Uni1, Uni2, Uni3, 3 models in Multi1 scenario, 18 models in Multi2 scenario), and (B) in the six MDs models of the Multi2 scenario.

**A**

|  | Selection of the 10 best families | | | Selection of the 20 best families | | | Selection of the 50 best families | | |
| --- | --- | --- | --- | --- | --- | --- | --- | --- | --- |
| Trait | Number of families selected at least once | Number of families selected in at least 12 out of the 24 models | Number of families selected in all the models | Number of families selected at least once | Number of families selected in at least 50% of the models | Number of families selected in all the models | Number of families selected at least once | Number of families selected in at least 50% of the models | Number of families selected in all the models |
| FL | 22 | 9 | 3 | 40 | 20 | 8 | 82 | 50 | 21 |
| PH | 28 | 8 | 2 | 47 | 21 | 2 | 94 | 49 | 19 |
| YLD | 45 | 8 | 0 | 82 | 15 | 0 | 142 | 51 | 1 |
| ZN | 35 | 8 | 0 | 58 | 20 | 1 | 119 | 48 | 9 |

**B**

|  | Selection of the 10 best families | | | Selection of the 20 best families | | | Selection of the 50 best families | | |
| --- | --- | --- | --- | --- | --- | --- | --- | --- | --- |
| Trait | Number of families selected at least once | Number of families selected in at least 50% of the models | Number of families selected in all the models | Number of families selected at least once | Number of families selected in at least 50% of the models | Number of families selected in all the models | Number of families selected at least once | Number of families selected in at least 50% of the models | Number of families selected in all the models |
| FL | 16 | 10 | 6 | 32 | 22 | 11 | 72 | 50 | 30 |
| PH | 15 | 11 | 5 | 26 | 23 | 11 | 68 | 54 | 34 |
| YLD | 15 | 11 | 6 | 32 | 18 | 10 | 67 | 52 | 33 |
| ZN | 18 | 11 | 3 | 38 | 18 | 11 | 79 | 51 | 28 |

Table S6a - Variance decomposition and broad sense heritability (H²) obtained using Model 2 by trait and generation.

|  |  | PCT27A S_0:2_ | | | PCT27A S_0:3_ | | |
| --- | --- | --- | --- | --- | --- | --- | --- |
| Trait | Variance component | Variance | Proportion | H² | Variance | Proportion | H² |
| FL | Genotype | 10.69 | 37.18 | 0.64 | 8.42 | 36.34 | 0.75 |
|  | Location:Genotype | 10.01 | 34.82 |  | 1.92 | 8.29 |  |
|  | Bloc:Rep:Location | 1.72 | 5.98 |  | 1.89 | 8.16 |  |
|  | Residuals | 6.33 | 22.02 |  | 10.94 | 47.21 |  |
| PH | Genotype | 24.38 | 32.54 | 0.71 | 22.25 | 39.18 | 0.77 |
|  | Location:Genotype | 7.57 | 10.11 |  | 4.96 | 8.73 |  |
|  | Bloc:Rep:Location | 4.54 | 6.06 |  | 4.45 | 7.84 |  |
|  | Residuals | 38.43 | 51.29 |  | 25.13 | 44.25 |  |
| YLD | Genotype | 2629 | 14.37 | 0.43 | 516 | 5.34 | 0.21 |
|  | Location:Genotype | 3201 | 17.49 |  | 1872 | 19.36 |  |
|  | Bloc:Rep:Location | 1394 | 7.62 |  | 1264 | 13.08 |  |
|  | Residuals | 11075 | 60.52 |  | 6015 | 62.22 |  |
| ZN | Genotype | 1.63 | 25.91 | 0.58 | 1.31 | 25.69 | 0.57 |
|  | Location:Genotype | 1.5 | 23.85 |  | 1.28 | 25.1 |  |
|  | Bloc:Rep:Location | 0.55 | 8.75 |  | 0.43 | 8.43 |  |
|  | Residuals | 2.61 | 41.49 |  | 2.08 | 40.78 |  |

Table S7– Predictive ability of the different scenarios and models (means ± standard deviation). For each trait, stars indicate models significantly higher than the Uni1 model.

| Scenario | Model | FL | PH | YLD | ZN |
| --- | --- | --- | --- | --- | --- |
| Uni1 |  | 0.251 ± 0.077 | 0.296 ± 0.068 | 0.301 ± 0.084 | 0.190± 0.081 |
| Uni2 |  | 0.323 ± 0.002* | 0.312 ± 0.004 | 0.267 ± 0.003 | 0.306 ± 0.003* |
| Uni3 |  | 0.297 ± 0.002 | 0.294 ± 0.003 | 0.239 ± 0.004 | 0.288 ± 0.003* |
| Multi1 | MM | 0.383 ± 0.078* | 0.222 ± 0.084 | 0.197 ± 0.101 | 0.269 ± 0.089* |
|  | MDs | 0.363 ± 0.074* | 0.278 ± 0.069 | 0.224 ± 0.082 | 0.220 ± 0.074* |
|  | MDe | 0.304 ± 0.082* | 0.254 ± 0.072 | 0.249 ± 0.077 | 0.172 ± 0.074 |
| Multi2 | Random_25_MM | 0.251 ± 0.019 | 0.231 ± 0.042 | 0.068 ± 0.071 | 0.174 ± 0.050 |
|  | Random_50_MM | 0.276 ± 0.015* | 0.261± 0.036 | 0.114 ± 0.072 | 0.207 ± 0.036 |
|  | Random_75_MM | 0.298 ± 0.009* | 0.303 ± 0.019 | 0.182 ± 0.042 | 0.255 ± 0.020* |
|  | Random_25_MDs | 0.282 ± 0.021* | 0.280 ± 0.018 | 0.128 ± 0.024 | 0.240 ± 0.027* |
|  | Random_50_MDs | 0.302 ± 0.018* | 0.301 ± 0.016 | 0.161 ± 0.021 | 0.270 ± 0.021* |
|  | Random_75_MDs | 0.318 ± 0.011* | 0.311 ± 0.008 | 0.176 ± 0.016 | 0.285 ± 0.016* |
|  | Random_25_MDe | 0.237 ± 0.051 | 0.192 ± 0.059 | 0.051 ± 0.071 | 0.136 ± 0.082 |
|  | Random_50_MDe | 0.254 ± 0.057 | 0.173 ± 0.076 | 0.057 ± 0.088 | 0.144 ± 0.073 |
|  | Random_75_MDe | 0.259 ± 0.042 | 0.193 ± 0.077 | 0.062 ± 0.082 | 0.154 ± 0.072 |
|  | CDmean_25_MM | 0.275 ± 0.010* | 0.203 ± 0.017 | -0.031 ± 0.034 | 0.162 ± 0.033 |
|  | CDmean_50_MM | 0.286 ± 0.008* | 0.244 ± 0.013 | -0.12 ± 0.030 | 0.192 ± 0.016 |
|  | CDmean_75_MM | 0.304 ± 0.004* | 0.298 ± 0.011 | 0.121 ± 0.020 | 0.278 ± 0.010* |
|  | CDmean_25_MDs | 0.316 ± 0.014* | 0.250 ± 0.010 | 0.128 ± 0.015 | 0.210 ± 0.018 |
|  | CDmean_50_MDs | 0.321 ± 0.009* | 0.282 ± 0.007 | 0.150 ± 0.012 | 0.253 ± 0.011* |
|  | CDmean_75_MDs | 0.326 ± 0.004* | 0.312 ± 0.006 | 0.173 ± 0.009 | 0.280 ± 0.008* |
|  | CDmean_25_MDe | 0.256 ± 0.043 | 0.173 ± 0.074 | 0.056 ± 0.075 | 0.117 ± 0.072 |
|  | CDmean_50_MDe | 0.270 ± 0.038 | 0.161 ± 0.075 | 0.067 ± 0.079 | 0.125 ± 0.072 |
|  | CDmean_75_MDe | 0.261 ± 0.051 | 0.168 ± 0.815 | 0.173 ± 0.009 | 0.121 ± 0.076 |


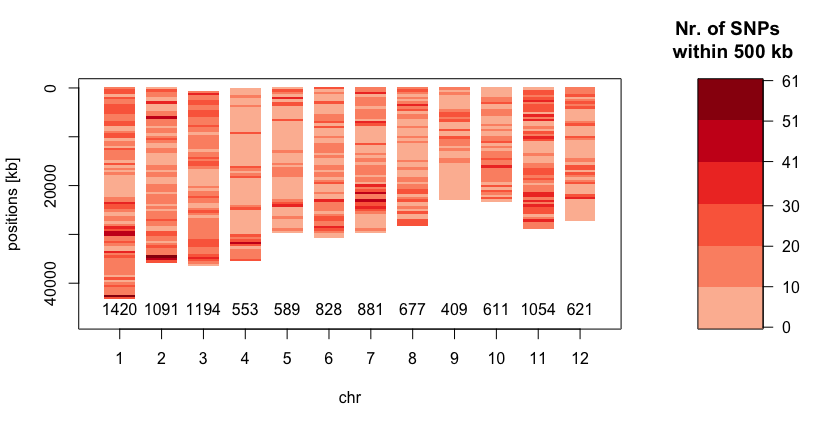


Figure S1 - Density of SNP markers in the two populations (PCT27A and PCT27B) and the temporal checks set (713 S_0_ plants) in the 12 chromosomes (chr) using the R package Synbreed (Wimmer et al. 2012).


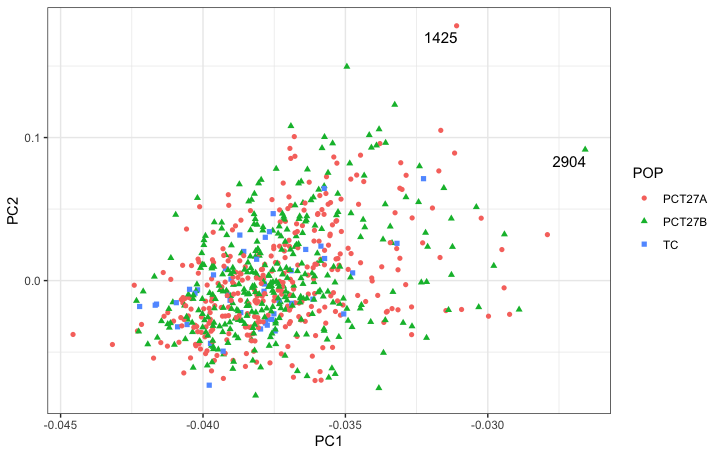


Figure S2 - Biplot from PCA performed on 7,766 SNP (after pruning) and 713 S_0_ plants (PLINK). Grouping by color of PCT27A, PCT27B and the temporal checks (TC belonging to PCT27A).
